# Enviro-Net: From Networks of Ground-Based Sensor Systems to a Web Platform for Sensor Data Management

**DOI:** 10.3390/s110606454

**Published:** 2011-06-17

**Authors:** Gilberto Z. Pastorello, G. Arturo Sanchez-Azofeifa, Mario A. Nascimento

**Affiliations:** 1 Department of Earth and Atmospheric Sciences, University of Alberta, 1-26 Earth Sciences Buiding, T6G 2E3, Edmonton, AB, Canada; E-Mail: gilbertozp@acm.org; 2 Department of Computing Science, University of Alberta, 2-32 Athabasca Hall, T6G 2E8, Edmonton, AB, Canada; E-Mail: mario.nascimento@ualberta.ca

**Keywords:** micro-climate monitoring, phenology monitoring, sensor networks, Web-based sensor data management

## Abstract

Ecosystems monitoring is essential to properly understand their development and the effects of events, both climatological and anthropological in nature. The amount of data used in these assessments is increasing at very high rates. This is due to increasing availability of sensing systems and the development of new techniques to analyze sensor data. The Enviro-Net Project encompasses several of such sensor system deployments across five countries in the Americas. These deployments use a few different ground-based sensor systems, installed at different heights monitoring the conditions in tropical dry forests over long periods of time. This paper presents our experience in deploying and maintaining these systems, retrieving and pre-processing the data, and describes the Web portal developed to help with data management, visualization and analysis.

## 1. Introduction

Monitoring ecosystems at high spatial and temporal resolutions still is a challenging endeavor. Satellite-embarked sensors that offer regular passes support only coarse resolution monitoring and on-demand high resolution satellite or airborne-based monitoring are still too expensive to be considered viable options for frequent data collections. Furthermore, validation of satellite and airborne measurements against the values observed at ground level is often difficult to obtain. Ground-based, or *in-situ*, sensor systems for environmental monitoring have associated challenges as well [1], but have undergone a considerable evolution recently. Such systems are now capable of collecting data at very high temporal resolution for very specific ecosystems through long periods of time. In particular, the use of wireless sensor systems has been shown to be very effective in this type of monitoring [2], from the cost perspective and increasingly in terms of performance and reliability as well.

There are many challenges associated with high resolution (both spatial and temporal) *in-situ* environmental monitoring, many of which already well recognized in the literature. Rundel *et al.* [1], for instance, discuss how these networks generate more data than can be managed by traditional methods for field research data, with data quality assurance and control surpassing capabilities of single individuals dealing with the data, but still being required to produce high-quality data. The large variety of problems impacting quality can be more easily detected by using adequate cyberinfratructure for automating the detection, which also allows more timely identification of problems in the deployments themselves. They also argue that, although data storage and retrieval is reasonably easy to attain, publishing and sharing data is not as straightforward. Still according to the authors, one of the advantages of this integrated approach for offering data from multiple sensors is the larger world view generated, which is not possible with single sensors—at least not at these spatio-temporal scales. The authors also acknowledge the importance of training scientists in using *in-situ* monitoring tools, the flexibility of power requirements for these systems (especially wireless) and the use of energy harvesting, problems related gaps in the data (from numerous causes), difficulty to assess precision and fidelity in such systems, and the value of commercial availability for decreasing costs and scaling up deployments sizes.

Hart and Martinez [3] discuss power management, large volumes of data and required cyberinfrastructure, beginning of commercial efforts, and data quality control as important issues concerning *in-situ* environmental monitoring. They also raise additional points that require more work, such as assessment of environmental conditions any equipment needs to withstand them (e.g., temperature, pressure, vibration); standardization requirements related to data and metadata representation; security requirements, preventing tampering with both equipment and datasets within the data management systems; and, better means for data interpretation (e.g., by using new methods for data mining). Another relevant effort can be found in the report from Estrin *et al.* [4], who focus on cyberinfrastructure. Key points include: the need for better prototyping and design of end-to-end test-beds to allow validation across wide ranges of environments, applications and domains; creation of better services regarding time synchronization, *in-situ* calibration, and adaptive duty cycling, among others; seamless use of high performance computing facilities for data processing; tools to improve support for metadata; and, collaboration efforts as a basis for training new scientists (from multiple domains) and as a mechanism for sustaining long term deployments.

This paper distills our experience in deploying and managing *in-situ* sensor systems within the Enviro-Net Project (http://www.enviro-net.info/). Currently, Enviro-Net includes 39 deployments spread throughout nine sites in six different countries (Argentina, Brazil, Canada, Costa Rica, Mexico and Panama), and is coordinated at the University of Alberta, in cooperation with local partner research teams at each site. The initial goal of the deployments was to monitor vegetation phenology, the study of climate effects on periodic biological activity [5], correlating it with environmental variables, such as availability of light, air temperature, *etc.* These and other variables are monitored by different types of sensing systems, with the collected data being transmitted back to Internet servers in Alberta either through a commercial satellite up-link or being manually retrieved from the data loggers and then sent via email, FTP or Enviro-Net’s website. The following gives but one example of the applicability and usefulness of such type of systems. From the data collected by a combination of two types of specialized solar radiation fluxes sensors, it is possible to derive different vegetation indexes, which can be used as proxies to monitoring phenological responses. In order to distinguish vegetation distribution, particularly from perspectives such as species distribution or successional stage, the areas to be monitored are numerous and relatively small. Similarly, short term effects of isolated climatic phenomena (e.g., a rainstorm or sharp changes in temperature) require higher rates of data acquisition. These characteristics require higher spatial and temporal resolutions only achieved through *in-situ* monitoring of each ecosystem.

In this context, detailed discussions of how we dealt with these challenges within the Enviro-Net Project form the main contributions of this paper, particularly considering the scenario under which the project was developed. The monitored sites are mostly tropical dry forests in remote locations, which are challenging environments for both equipment performance and personnel’s ability to work. Also, all deployments are based on inexpensive and commercially available technology, essential characteristics to allow scalability and reproducibility of experiments. The heterogeneity of equipment from different manufacturers also introduce difficulties, mainly regarding systems maintenance. Having long term (multiple-year) deployments impose extra management requirements. Integrated data management, a fourth aspect, presents numerous challenges ranging from data quality control to user interface usability. Finally, and maybe the most relevant aspect, is the issue of high spatial and temporal resolutions, considered not only within a single deployment, but also among different deployments both in the same and different sites. Part of these challenges have simple individual solutions, however, from a more holistic perspective, the integration of the solutions for all of them is what actually enables the use of sensors systems for *in-situ* environmental monitoring. After a review of related work on Section 2, this paper describes our solutions regarding deployments of *in-situ* monitoring systems in Section 3, pre-processing and treatment of data in Section 4 and data publication and accessibility using a Web-based system in Section 5.

## 2. Related Work

This section divides related work discussion into applications (covering the motivation for *in-situ* monitoring), deployments (showing experiences in installing and maintaining sensor systems), and data management (comparing different efforts in dealing with the large amounts of sensor data generated).

### 2.1. Applications

Environmental monitoring is one of the driving forces behind the adoption of ground-based sensing systems, pushing the need for higher spatial and temporal resolution. Examples of efforts in this direction include: (i) the creation of the National Ecological Observatory Network (NEON) [6], which aims at studying climate change, land-use change and invasive species on a continental scale using, among other methods and technologies, ground-based deployments of sensor systems; (ii) FLUXNET [7], which use micrometeorological and flux towers to measure exchanges of carbon dioxide, water vapor, and energy between terrestrial ecosystems and the atmosphere. These initiatives heavily rely on long term ground-based monitoring solutions. FLUXNET has a public data management framework called Fluxdata.org [8], which also offers flexible metadata support. However, due to the diversity of equipment and protocols for deployment and data pre-processing, data integration within the Fluxdata.org system is limited, mostly offering access to data on the original format provided by the data producers. This limits the possibilities of applying filters and aggregation operations to the data or generating derived data products within the system. Although our system also deals with a variety of equipment, the deployment protocols are largely uniform, and pre-processing protocols are developed using a centralized approach, which allow us to achieve a considerable level of data integration within Enviro-Net.

These and other initiatives, aiming at integration of ground-based monitoring efforts, are leading to an evolution from single site environmental monitoring into networks for environment observation [3]. This evolution culminates with the current vision for a *Sensor Web* [9–11], encompassing several types of deployments of sensor systems, interconnecting them globally through a Web-based integration strategy using standards developed by the Sensor Web Enablement (http://www.opengeospatial.org/projects/groups/sensorweb) Working Group of the Open Geospatial Consortium, Inc. (OGC) (http://www.opengeospatial.org/).

A small clarification on the definition for (wireless) sensor networks may be in order. Mainly within Computing Science (CS) research [12,13] and in earlier Sensor Web related efforts [9], this definition is narrower than what is used in this paper. In this more restrictive definition, a (wireless) sensor network is based on nodes (also known as “motes”) that have sensing, data storage/processing, and communication components plus a power source. These nodes are usually autonomous and operate cooperatively—by communicating amongst themselves—to collect and process data, also being programmable, *i.e.*, able to behave differently according to, for instance, the type of application, power supply conditions, environmental conditions, *etc.* Although we have used this type of wireless nodes in our deployments, we do not require the capability of offering communication amongst network’s components. Instead, we adopt the centralized type of processing architecture as classified by [12], being more in line with the current Sensor Web approach to networks [11]. It is sufficient for us, for instance, that the connection of sensing elements be done at the level of integrated data products.

Applications of *in-situ* monitoring systems are also the topic of other research efforts. Porter *et al.* [2] present a good review of the capabilities of wireless sensor networks (WSN) to be applied within the ecological domain. Hamilton *et al.* [14], while covering capabilities of networks of sensors applied to ecology as well, also highlight the idea of ecological observatories, adopted within NEON. An extensive review of *in-situ* monitoring efforts is presented by Rundel *et al.* [1], classified according to their area of focus: above ground, under-ground, and aquatic environments. Porter *et al.* [15] discuss the state of the sensing technology, what can already be accomplished and a few areas that require more development (e.g., data management software and new types of sensors). Precision agriculture is a particularly relevant application area for pervasive sensing technology. For instance, Lee *et al.* [16] evaluate monitoring applied to specialty crop, while Matese *et al.* [17] use wireless sensor network in vineyard monitoring, and Aquino-Santos *et al.* [18] evaluate data transmission protocols in small scale deployments in watermelon fields. In this paper, we discuss aspects that apply to many of these scenarios, particularly if considering them in a long term monitoring perspective. However, our focus is on practical and logistics aspects of deploying and maintaining equipment, retrieving and managing the data, and supporting analysis of data products.

### 2.2. Deployments

Other research groups have discussed their efforts with ground-based deployments of sensor systems, mostly focusing on the use of wireless equipment. A pioneering effort in applying wireless sensor networks was the habitat monitoring experiment in the Great Duck Island [19] in the coast of Maine in the United States, deployed to offer a less intrusive way to study behavior and nesting of seabird colonies. The SensorScope project [20,21] is another example, taking place mainly in Switzerland. They have described their experience with developing the hardware and software for their wireless system, performing tests, and going on deployment expeditions, along with their architecture and communication protocols. With a focus on solar energy availability, AdaptSens [22] adopts system-wide levels of operation to cope with different amounts of available energy. GreenOrbs (http://www.greenorbs.org/) [23] is a long term effort for monitoring an university campus urban forest close to Hangzhou in China, using a large number of nodes. LUSTER [24] is a system for monitoring ecological variables that implements fault-tolerant distributed storage over a delay-tolerant network using an hierarchical architecture; the system also covers user interaction both in the field expeditions and a web interface for data retrieval. Another effort [25], aiming at monitoring the UNESCO World Heritage site Mogao Grottoes in Dunhuang, China, implemented a low power wireless monitoring system inside the site’s caves with a tailored long distance connection to transmit the data back to an on-line server. Another World Heritage site, a rainforest ecosystem in Queensland, Australia, was monitored by a wireless sensor network project [26], which served as a prototype for future long term deployments using similar configurations. Another interesting application, monitoring the activities of volcanoes in Ecuador [27,28], entails addressing issues such as higher sampling rates (100 Hz or more), need for higher accuracy and more expensive sensors. Changing the spatial scale a little, monitoring a single redwood tree [29] in California in the United States, offered new insight in understanding the microclimate surrounding this type of tree. Reports on deployment experiences also focus on the diversity of problems faced when using wireless sensing equipment, such as the LOFAR-agro project [30] that experienced problems ranging from hardware failures to network protocols errors and software problems. While deployment related efforts in our work focus on issues related to managing the life cycle of ground-based sensor data, other works [31,32] bring evaluations of technology for wireless sensor network equipment, including communication protocols, power consumption and data transmission issues.

To the best of our knowledge, none of the deployment efforts reviewed here address the same scenario as ours: having (multi-year) long term deployments, based on cooperative efforts of several (heterogeneous) teams, using commercially available equipment from multiple manufacturers, with an integrated effort of data retrieval, quality control and data availability through an easy to use Web-based platform. We believe this is a more realistic scenario for ground-based environmental monitoring efforts. The current efforts within the Life Under Your Feet project (http://lifeunderyourfeet.org/) [33] are the closest to our own, also having long term, spatially distributed deployments with a Web-based data visualization interface integrated with geolocation information. However, they do not seem to deal with heterogeneous equipment and data formats, nor offer filtering/aggregation options, derived datasets or quality information in their data management solution.

### 2.3. Data Management

Many of the challenges related to sensor data management have been known for a while [4]. However, several technical and non-technical questions still remain unaddressed. Broad scope projects for management of earth observation data try to present a top-down approach to data management. One such project is DataOne (http://www.dataone.org/), an effort towards distributed cyberinfrastructure for Earth observation data, bringing together a multitude of data providers and consumers. Another effort is our partner project GeoChronos (http://www.geochronos.org/), which implements means for sharing (and interacting with) tools, datasets and libraries of records within the Earth observation domain. Enviro-Net, however, uses more of a bottom-up approach, offering specialized solutions for the types of data supported, expanding these types as needed. This allows data management solutions that are geared towards specific needs to answer specific science questions.

Although it is common to think about sensor data management as stream data management, with the associated challenges (on-line aggregation, classification, *etc.*) [34], at least within environmental research, particularly in ground-based monitoring, this is not a frequent scenario. Most of the current applications based on sensor data use the perspective of historical (or an archive of) time series data. Applications using the stream data perspective are only beginning to appear, and the current applications that do require that perspective—e.g., volcano monitoring [27]—are still the exception. Data manipulation for most of the current applications is done after having the data collected and stored, applying a variety of analytical operations in an offline fashion [8,35].

Middleware software for automating control of deployments is also the focus of current research efforts, in form of architectures for integrating different network deployments [36], or Web-based interfaces for interaction with and control of wireless deployments [37]. Our focus, on the other hand, is on managing the data products rather than controlling the equipment from within our system.

The data archival aspect of data management involves not only storage of data, but also retrieval, documentation, access control, among other issues. Furthermore, data curation of long-term repositories involves not only handling the data but also helping scientists answering research questions and also maintaining the underlying computational infrastructure [38]. Within Enviro-Net, although we are only beginning to to devise our long term plans for infrastructure maintenance, our system already offers data access with a number of flexibility aspects to foster efficient use of the data. Efforts on applying digital library practices in support of sensor data management are also gaining acceptance [39]. Issues of data quality and integrity, as well as the elements of data collection that affect them, need to be an integral part of such efforts [40], particularly from the perspective of making data documentation available along with the datasets. In this scenario, metadata becomes as valuable as the datasets themselves, from quality metadata about deployments [41], to offering search and annotation options and enriching visualization [42]. Finally, Application Programming Interfaces (APIs) allow data to be accessed in a programmatic way, which can be achieved, for instance, using Web services interfaces (using Sensor Web Enablement standards) or using specialized solutions such as a wrapper-based middlewares [43] or REST-based APIs [44]. Data quality aspects are an integral part of Enviro-Net, and are being improved, particularly regarding documentation and metadata coverage. Although data ingestion is largely automated and data access is possible through the Web user interface within Enviro-Net, data access using a programmatic interface is still under development.

## 3. Sensor Systems Deployments

Apart from a few test installations, all of our deployments are intended to be long term, collecting data for a minimum of two to three years. The earliest deployments were installed in mid 2007, with the first wireless deployments installed in mid 2008. All deployments suffered from interruption in data collection on some scale, usually from a few days up to a couple of months, depending on how early the problem was detected. Earlier deployments suffered 100% failure rate due to equipment design being incompatible with tropical environments. Many problems were related to unexpected interactions of environmental conditions with the equipment. However, most of the deployments are still operational today, with secured funding for maintaining them operational until at least 2013.

Currently, Enviro-Net has 39 permanent deployments, plus temporary deployments in Edmonton, Canada for equipment testing and calibration. The *Biosphere Reserve of Chamela-Cuixmala* in the state of Jalisco, Mexico has a tower (overlooking the top of the canopy) and a wireless understory sensor system. The number of nodes in a wireless deployment is usually 12, but there are deployments with as few as five and as many as 20 nodes, each node having between three to six sensors each. The *Santa Rosa National Park* in Costa Rica hosts two more towers. The *Parque Natural Metropolitano* in Panama has the most recent deployment with 24 thermocouples monitoring leaf temperatures. In Brazil, three sites have deployments: the *Mata Seca State Park*, the *Serra do Cipó National Park*, and the *Environmental Protection Area of the Pandeiros River*, all located in the Minas Gerais state. The Mata Seca park hosts five towers and eight understory deployments (including four wireless deployments), all in the *cerrado* ecosystem, which is similar to a savanna; three understory deployments are active close to the Pandeiros river, also a *cerrado* ecosystem; and, Serra do Cipó park has five towers plus seven understory deployments, two of which using wireless systems, covering natural grasslands and forest vegetation in the *cerrado*. Finally, three deployments are operational in the province of San Luis in Argentina, a phenology tower monitoring a grassland ecosystem, and one tower and one wireless understory deployment installed in a adjacent *chaco* ecosystem. Two more wireless towers are operational *chaco* and grassland ecosystems in the province of Córdoba, Argentina. Three more deployments are expected to start data collection in 2011 in the province of San Luis. Although the ideal spatial scales for many applications requires higher numbers of nodes deployed to be considered high spatial density—more in line with our plans for future sensor networks—the intermediary step with 5–20 nodes per deployment was necessary to prove this kind of scale is feasible in remote locations with long term deployments. These are, however, dense enough to characterize many ecosystem level behavior (such as response to climatic events), and even differences between neighboring ecosystems. The experience acquired in these smaller deployments, which is the fundamental contribution of this text, serves as a basis for these larger scales expeditions.

The main challenge of having deployments across an entire continent is without question maintaining them. Partnerships with research groups based closer to the deployment sites proved essential, with the added issue of offering training to the people performing basic maintenance. The small amounts of time available for training leads to the choice of equipment that is simple to use and maintain. Hands on experience has proven to be the most efficient method to train new users, specially when focusing on how to deal with common problems. Special attention needs to be given to data retrieval and manipulation methods in order to allow tracking of data problems later in the processing chain. Documentation of our own group’s deployment protocols and data handling procedures complemented and helped with equipment manuals and specifications.

Regularity in systems maintenance is key in keeping them running within long term deployments. Life expectancy and calibration deviation for sensors are usually a parameter specified by the manufacturer. Enviro-Net deployments usually have two maintenance cycles: one for basic overall system check (and data retrieval for off-line deployments) and another for complete verification of the equipment. The basic cycle has intervals ranging from two weeks to two months, depending on the accessibility of the site and regularity of visits for other purposes. This task is usually performed by a member of the local research teams and involves cleaning the sensors if needed—mostly from dust build-up or obstructions such as leaves, insect or bird nests, etc–verification of the general health of the system, and data retrieval, usually the most relevant part in a basic maintenance cycle. The complete cycle intervals ranges from 6 to 12 months, and allows detection of a broader range of problems—e.g., battery charge retention capacity. This task is usually performed with one more experienced technician.

### 3.1. Sensors and Loggers

Tables 1 and 2 list the equipment used in our deployments. For datalogger systems, shown in Table 1, wired and wireless systems are available. In wired systems all the sensors are connected directly to the data logger and the communications with it are done mostly through a physical connection using a cable (serial or USB, for instance) to connect to a laptop. For wired deployments, we mostly used *Onset Computer Corp.* (http://www.onsetcomp.com/) data loggers; specifically the *HOBO Micro Station*, the *HOBO U12 Series* and the *HOBO U30 Series* models were employed.

Wireless systems, on the other hand offer different strategies to eliminate the need for cabled connections. As an example, the equipment manufactured by *Olsonet Communications Corporation* (http://www.olsonet.com/) offers two types of nodes: a collector and an aggregator. The former is connected to the sensors and is responsible for wirelessly transmitting the readings to the aggregator, which works as a centralization point for the data collection also dubbing as a short term data logger. The aggregator, however, requires a cable connection for setup or data recovery. A different strategy is used by the equipment manufactured by *MicroStrain, Inc.* (http://www.microstrain.com/), where each *ENV-Link^™^* node works as an individual data logger, but the connection to these nodes for setup and data retrieval is done through a wireless connection.

The storage capacity for samples in both types of loggers usually match the power consumption characteristics to achieve similar longevity in field deployments. As discussed later in this section a satellite up-link and a continuous battery recharging capability (e.g., using solar panels), would allow even longer time spans. However, since in practice maintenance is necessary long before these limits are reached, battery and storage lifetimes are not a limitation for most of these types of equipment.

The biggest advantage of wired equipment is reliability, being in use longer, and tested under many combinations of conditions. Besides limited spatial coverage, when compared to wireless systems, the most problematic aspect of this technology is accessibility. Everything requiring a physical connection between the logger and the laptop with the control software, having to climb up a tower to perform tasks as routine as retrieving data is a somewhat serious limitation. Even using longer cables for the sensors, which still have a limited maximum length on account attenuation of the electric signal, towers for higher canopies require climbing to access the logger.

For environmental monitoring, the major advantage of wireless sensor systems is the possibility of covering larger areas, without giving up high spatial and temporal resolution, and at a reasonably low cost. One low point of the technology is that it is still fairly new as a commercial product, and still needs some adaptation. Errors in communication protocols, radio range limitations, power management related issues, lack of features in the control software packages, and breaches in weather proofing cases weight in at the cons for wireless systems. However, our experience shows the technology has already reached the tipping point to becoming viable for use in long term, harsh environment deployments.

Commercial availability of *wireless sensor networks* (WSN), as a technology, is still limited. Although the original ideas for WSN—*i.e.*, large number of general purpose nodes distributed in very dense deployments, randomly placed, almost weightless, and disposable—have yet to materialize [20,28,45], wireless technology used in conjunction with sensory equipment is proving to be invaluable in monitoring larger areas at the scale of a single ecosystem.

Table 2 lists the main sensors use in our deployments, which are are well known, commercially available, inexpensive, and based on established technologies. With the variables listed, it is possible to extract plenty of derived information from them, such as vegetation indexes and light absorption patterns for photosynthesis. In our deployments, we used solar radiation sensors provided by Onset and *Apogee Instruments, Inc.* (http://www.apogeeinstruments.com/); air temperature and relative humidity sensors by *Sensirion Inc.* (http://www.sensirion.com/) and Onset (The Onset temperature and relative humidity sensors used are repackaged Sensirion sensors); and, soil moisture sensors by *Decagon Devices, Inc.* (http://www.decagon.com/) Lower cost sensors systems usually do not offer calibration options for the user; they have their calibration adjusted at the manufacturer (who usually offer recalibration services).

### 3.2. Deployment Configurations

Within the Enviro-Net Project there are currently two main types of deployments: phenology towers and understory installations. A phenology tower uses two solar radiation flux sensors (also called pyranometers), measuring wavelengths between approximately 300 to 1,100 nm, and two Photosynthetically Active Radiation flux sensors (or PAR sensors), which measure wavelengths between approximately 400 to 700 nm. Ratios of these measurements can be used to derive vegetation indexes such as Normalized Difference Vegetation Index (NDVI) or Enhanced Vegetation Index (EVI)–see, for instance [46–49]. Such indexes can be used as proxies to monitor vegetation phenology. Understory deployments are used to monitor the conditions below the canopy level, and usually cover a larger area.

**Figure 1.**
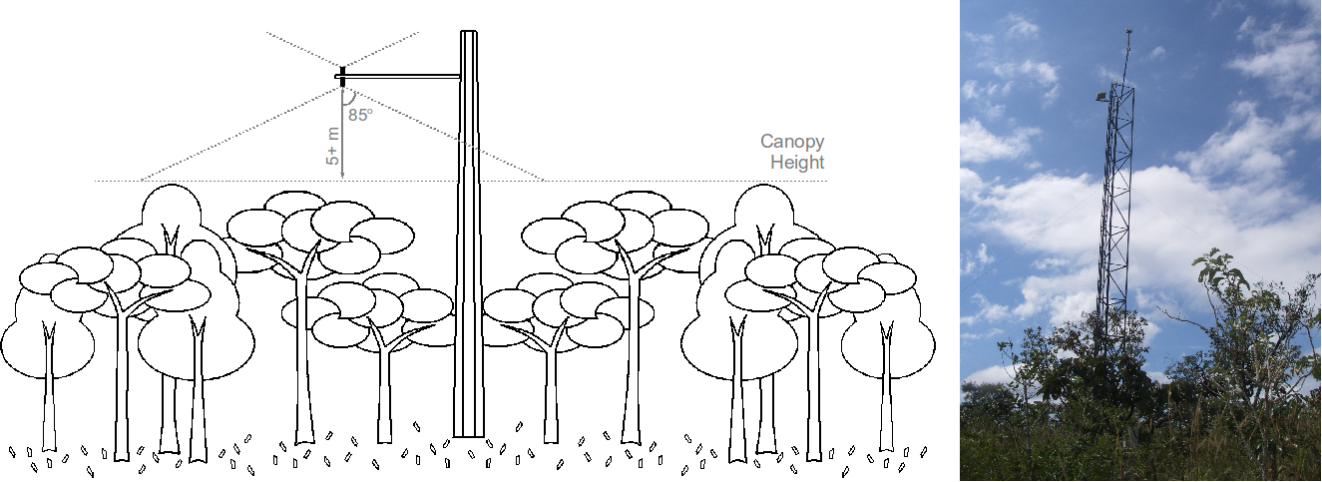
Phenology tower schematics (left) and a tower in Brazil (right).

Figure 1 shows the schematics of a phenology tower on the left, with two PAR sensors and two pyranometers, one of each measuring incoming solar radiation and one of each measuring reflected solar radiation. The right side of the figure is a photo of one phenology tower installed in Brazil, which raises the sensors eight meters from the ground, six meters above the canopy.

Most radiation flux sensors have view angles of up to 85° from zenith (when oriented up, *i.e.*, measuring incoming radiation) or nadir (when oriented down), with a uniform 360° rotation. With that, the radius that affects the readings is up to around ten times the distance (*h*) between the sensor position and the surface being monitored (*i.e.*, *radius* = tan(85°) ×*h*). For our deployment, we usually have at least five meters between the top of the canopy and the sensor measuring the reflected radiation (8 to 15 m in total), leading to a coverage radius of at least 50 m in the monitored area.

Obstructions within the range of a sensor interfere with the reading and might not be easy to identify from the data only—e.g., higher canopy of adjacent ecosystems or a nearby tower with other instruments may interfere with sensor measuring incoming radiation. A sensor measuring radiation reflected from the canopy is more susceptible to interference—e.g., the positioning of solar panels, whose reflectiveness greatly affect readings. Large panels should be positioned outside of the interference radius, while smaller panels can be positioned at the same height as the sensor for no interference. Note that it is difficult to position radiation sensors and solar panels at different orientations, since both should use the optimal exposure angle to the sun, roughly North, in southern latitudes, or South, in northern latitudes.

Monitoring the conditions under the canopy level, *i.e.*, understory deployments, allows assessing a different range of micro-climatic conditions and also soil condition—e.g., temperature and moisture levels. Understory deployments are usually easier to access, and with that, they are useful for validating the readings observed in a tower and also as a backup for certain variables in case of sensor malfunction in a tower. Using wireless systems substantially increases the spatial coverage of understory deployments with a fraction of the increase in cost and efforts to retrieve data and maintain the system.

**Figure 2.**
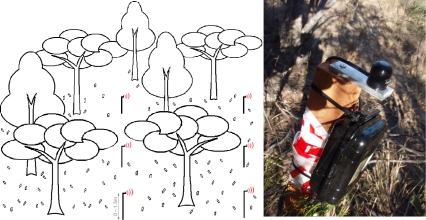
Understory schematics (left) and a node in Argentina (right).

Figure 2 depicts an example of such a wireless deployment on its left side. On the right side, it shows a node deployed in the *chaco* ecosystem in Argentina. The height at which the sensors are installed in this case is also determined by the canopy’s height, usually ranging from right on the ground (e.g., for grasslands) to 1.5 m for taller canopies. One example of application that relies on the spatial coverage and resolution of understory wireless deployments is deriving Leaf Area Index (LAI)—see [48,50], for instance. LAI, along with Plant (PAI) and Wood (WAI) Area Indexes [51], are important indicators of vegetation productivity, being also used as a reference for crop growth rates. Combining readings from a phenology tower with understory readings of absorbed solar radiation fluxes, it is possible to derive NDVI for the location of each node. Using NDVI and knowing an appropriate conversion factor, characteristic to each ecosystem, it is possible to calculate LAI for each node [48]. This allows the creation of maps of very high spatial and temporal resolutions for both NDVI and LAI.

**Figure 3.**
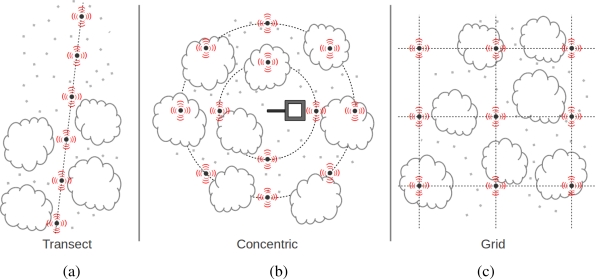
Deployment strategies: (**a**) transects; (**b**) concentric circumferences; and (**c**) grids.

Having the option of deploying a large number of sensors in a given area also raises the question of how to distribute these sensors. We have adopted three different strategies to spatially distribute nodes and their sensors. Figure 3 illustrates these strategies. The first approach, shown in the left, is intended to monitor a linear region along a transect. This is particularly useful for monitoring transitions between ecosystems or exposition to different conditions within the same ecosystem. The center of the figure shows distribution of nodes in concentric circles, which is sometimes called a “star” deployment. This type of deployment is used mostly to monitor conditions around a point of interest, usually corresponding to the footprint of phenology or carbon flux towers, allowing combination of measurements from both deployments. A third strategy is to deploy nodes in a grid, covering a potentially larger area of interest. Regularly spaced grids are useful for uniform monitoring throughout an area. However, irregular grids can also be useful when special conditions occur within a region of interest. Examples include part of an area that is also being monitored by other experiments (e.g., leaf collection for chlorophyll measurements); or patches affected by fire and monitoring their recovery is of interest.

### 3.3. Deploying Sensors Systems

From a logistics perspective, installing tower and understory systems have fairly different characteristics. Phenology towers reached up to 15 m in one of our deployments, with 9 m being the most common height. Selecting the location for installing a tower that high must take into account the representativeness of the ecosystem, the impact of building it, and the accessibility to bring its parts to the site. Another important issue is the uniformity in the height of the canopy. Too much variation in the tree heights will lead to scaling problems in the data, an area with taller vegetation will be contributing significantly less to the readings. When installing a phenology tower intended to be used in a long term data collection, the growth of the vegetation should also be taken into account. Younger ecosystems might grow considerably at intervals as short as one year, forcing height upgrades to a tower.

The height of the canopy is also a concern for understory deployments. Ecosystems with lower canopies, such as grasslands, require that solar radiation flux sensors be positioned almost adjacent to the ground, while taller canopies allow sensors in a higher position (0.60 to 0.10 m are common heights). For wireless deployments, the node is usually installed in a higher position to improve radio signal range, while the sensors are deployed at the appropriate heights.

Although it might seem like a trivial task at first, correctly positioning the sensors should take into consideration a number of factors. One issue is the creation of unnatural sources of shade (e.g., from the pole where the node sits) into the sensor. For deployments in the northern (southern) hemisphere, positioning radiation flux sensors South (North) of obstructions avoids this issue. Air temperature and relative humidity sensors are also affected by their positioning. Besides being hosted at solar radiation shields and being positioned as to allow for air circulation, they should also keep some distance from radiation absorbing materials. Most of the weatherproofing cases, for instance, absorb non-negligible quantities of solar radiation. We had cases of temperature deviations of up to 20 °C because of a dark weatherproof case.

One crucial aspect to sensor systems deployments in tropical ecosystems is the exposure to constantly high relative humidity. Values between 90%–100% are common in these environments. Combined with high temperatures, this condition transformed many weatherproof casings into humidity traps. The main problem was actually the difference of internal and external pressure in the cases. That made previously air tight cases absorb humidity while balancing the pressure, exposing the internal circuits and connectors. Both for loggers and sensors, even cases designed and tested to work underwater were susceptible to this problem. Adopting pressure relief valves significantly attenuated the problem, even though sometimes they can get clogged with dirt and stop working. Another adopted practice that also helped reduce this problem was to use silicone-based adhesives to seal borders and openings, around sensor cables and also around the sensors themselves.

For wireless sensor systems, testing the range of the radio system at the actual deployment site is essential. Vegetation distribution and terrain contours are difficult to predict beforehand and have a significant impact in the radio range. Two major aspects have shown to be of particular relevance when conducting this kind of test. Firstly, if the type of batteries used decrease the voltage offered to the system with time, the tests should not be conducted with new batteries. A more accurate test of radio range is achieved using more realistic battery levels—e.g., levels of battery similar to when a deployment running for more than half of the expected battery life. In case of rechargeable batteries, the charge level used should be the average level the batteries would have when going without charge for the maximum foreseeable period. For tropical dry forests the maximum period without non-negligible sun light exposure for charging batteries through solar panels is around two days. It is worth of note that regular alkaline (zinc and manganese dioxide) batteries, widely adopted to power nodes in sensor networks, do change their voltages depending on their level of charge.

The second aspect interfering with radio range is related to the vegetation density, particularly to changes in density throughout the seasons. Radio range is greatly affected by branches and leaves in the line of sight of the signal. Ranges of up to 300 m in a level and open field can be reduced to as little as 15 m (a factor of 20 reduction) simply by having a somewhat closed vegetation. In particular, radio frequencies at 2.4 GHz are severely attenuated by trees and leafs. This frequency is adopted by several wireless sensor systems, including the ZigBee Alliance (http://www.zigbee.org/) communications protocols (based on the IEEE 802.15.4 Wireless Personal Area Network standard), widely used in these systems. Furthermore, it is very usual for deployment campaigns to take place in the dry season, when rainfall is less of a concern for the schedule in deployment plans. However, foliage of deciduous vegetation can be at much lower levels than it will be in the wet season, which can significantly affect the range of radio signal. There is no definite solution to address the vegetation changes, since simulating the conditions of a different season is difficult. Monitoring the overall network health, which can be done in its simplest form by detecting gaps in the data, and repositioning nodes when necessary has been the best measure to address this issue in new ecosystems. These, in turn, serve as a reference for future deployments in similar ecosystems.

Seasonal change also can have an unexpected impact in the visibility of nodes and sensors. When installing sensors in the dry season, there are few obstructions and less color variability on the landscape. This makes visibility reasonably good. However, areas that significantly change their vegetation coverage or areas that have dense vegetation can become quite challenging from the point of view of visibility in the wet season. Using colorful markers—red or yellow ribbons or paint are effective for this—can save a lot of time when trying to find nodes and sensor that have been deployed for a while. Not relying solely on the GPS to locate small pieces of equipment such as individual nodes and sensors can be the difference between returning to base camp before or after sunset. One aspect of using such markers that was not taken into account in this work is the increased attractiveness color makers might exert on animals (particularly insects).

### 3.4. Retrieving Data

Data from ground-based sensor systems can be retrieved either *in-situ* or remotely. The former involves expeditions to the deployment sites, which can be very expensive. However, if the site is already being visited in a regular basis for other reasons (collecting leaf samples, for instance), this might become more feasible. Most of our current deployments are working in this scenario. This has proven to be quite an advantage from the perspective of maintenance of untested systems, allowing early detection of problems with equipment. With equipment proven to work well, using a remote solution is probably more cost and time effective.

Collecting data remotely might be achieved in a number of ways. One possibility is using a dedicated long range wireless communication system—e.g., by using a WiFi connection with repeaters—to transmit data at regular intervals to a computer installed in a location with permanent power supply. If there is also Internet connectivity, the data can be forwarded to on-line permanent archival systems. This alternative usually has a significant overhead of maintaining the local computer and the long range communications system running.

Another alternative is to use cellular networks with data capabilities. Although cellular coverage is not good in more remote areas, some regions have enough connectivity to allow data transmission in a fairly regular fashion. Using higher gain antennas improves signal reception, but the system must be prepared to go through reasonably long periods with no connectivity, preserving all data for delayed transmission. Since an actual Internet connection is provided with a cellular connection, the data can be transmitted directly to on-line archival servers.

A third type of remote data retrieval solution involves using a satellite up-link. This approach is also subject to communications failure (e.g., if there is too much cloud coverage). The connectivity provided here usually is not to the Internet, but connectivity to a service provider that receive the data from the satellite. This provider in turn makes the data accessible, often offering automated ways of retrieving the data from their on-line servers. In our case, systems that have proven to work consistently well have been equipped with a satellite transmitter.

Remote connectivity allows not only automated data retrieval, but also some level remote operation of the equipment. Options of stopping and starting the logger, setting sampling and storage rates are often available. In a few cases, it might be interesting to be able to set other parameters remotely, particularly with wireless systems. Research projects have explored configuring deployments remotely [37], even reprogramming loggers and collection nodes in some cases [20,36]. This level of flexibility in remote deployment configuration, however, is not yet commercially available.

## 4. Data Pre-Processing and Cleaning

When considering the volume of data generated by current sensor systems, automation of data management related tasks within a proper computational infrastructure is of paramount importance [1,3,4,10,33,52]. However, actual datasets generated by sensor systems might present a variety of problems and exceptions, which are often difficult to foresee. This is a severe drawback in attempts to automate the first data management phase: ingestion of data into any computational data management system. This sort of problems are often dismissed as being “implementation details”, but their implications can actually affect data quality parameters and models to store and distribute the data. In higher end (expensive) and/or homogeneous equipment this sort of problems are usually easier to tackle. However, in a setting like ours, using equipment from different manufactures, in a highly distributed effort, with an aim at low limits for equipment and maintenance costs, these issues are fairly commonplace.

The implementation of solutions for problems with raw datasets are usually carried out within a *data pre-processing* (or *data cleaning*) phase. Although these terms usually encompass explicit data quality verification or removal of erroneous readings (e.g., values outside the scale measured by a sensor), this section only considers problems that actually prevent (or are difficult to trace after) the ingestion of the data into a data management system. When compared to classification scales usually adopted in describing Earth observation data products, after the corrections in this section, the dataset should treated as “raw” data, or, as being at Data Processing Level 2 in the National Research Council (NRC) Committee on Data Management and Computation (CODMAC) [53] classification, or to Data Processing Level 0 used by NASA (http://science.nasa.gov/earth-science/earth-science-data/data-processing-levels-for-eosdis-data-products/). The next paragraph discuss the problems we had to handle when preparing our datasets.

### 4.1. Synchronization

Keeping correct temporal information for timestamping readings from distributed sensors can be really challenging, not to mention correcting time deviations after recording the data [54]. Time synchronization is an issue both at single deployment, with multiple collectors and/or loggers, and across deployments. Within a deployment, hardware imprecision and heterogeneous initial synchronization methods are the two main causes of synchronization problems. Time keeping in electronic equipment is based on crystal oscillators, which can deviate from their standard frequency with environmental conditions, especially temperature. This causes the time measurements to deviate as well, and affects almost all types of data logging equipments. In this case, the error is proportional to the sampling rate, which for applications such as seismology, with high sampling rates are, these errors are quite significant. For long term environmental monitoring, this can also be a problem. One solution is to have an accurate reference time keeping and a mechanism to keep the synchronicity among loggers. Possible solutions include having more precise equipment kept at a less exposed location or using GPS time as references. A few wireless communication protocols have time synchronization features embedded within their message exchanging mechanism [55].

When dynamic time synchronization against a reliable reference is not feasible, the initial synchronization method is the basis for all time information within a deployment. This is the most common scenario for our current deployments, with the usual mechanism for synchronization being based on the time information from the computer with the control software used to start a deployment. Therefore, the time information in that computer should be synchronized (e.g., by using Network Time Protocol, IETF RFC 5905 (http://tools.ietf.org/html/rfc5905)).

Data comparison from different deployments at small temporal resolutions must take into account potential synchronization errors. However, since sampling rates are commonly higher than desired temporal resolution, most data analysis is done with aggregated data instead of the entire dataset, which attenuates the effects of the time synchronization related errors, particularly when looking at hourly or even daily averages.

Similar to other reports [54], we also experienced power source related synchronization problems. Time measurement in some logging equipments can be affected by power outages or low voltages from the power source. Some types of equipment use the main power to keep time measurement running and, although time measurement usually requires very little power, if the supply is interrupted, the equipment’s clock gets reset.

Current data logging equipment and control software offer poor support to address time synchronization problems. Many of them don’t even let the user see what is the current time in logging system to manually check for time drifts. But this is evolving in control software for wireless systems, since these suffer more noticeably from time related problems.

### 4.2. Time Zones

When dealing with deployments that are geographically distributed throughout various timezones, establishing the correct local time can become an issue. Once again, relying on a computer’s time as a reference to timestamp the readings is a major cause of errors. Different versions of operating systems have different levels of automation regarding time zones and daylight saving time configurations, often allowing users to change these manually. Therefore, besides having the correct time on the computer, as already discussed, wrong configurations of time zone and changing configurations for daylight saving times can also lead to inconsistencies such as: having data for a single deployment timestamped with different daylight saving times, or difficulties determining which is the correct local time when comparing data for deployments in different time zones.

For our deployments, when issuing field laptops, time configurations always adopt the local standard time for the site, disabling automatic changes to daylight saving time. However, even rugged field laptops fail, and temporary misconfigured replacements can be used. Or, an even less elaborate problem, which happens often, new users get confused by seemingly “wrong” time settings and change the time configurations.

It is possible, however, to check time zone and daylight saving times against sun time. This is done by comparing several days of sunrise time from data collected by solar radiation sensors to expected sunrise times for the location. This method is not accurate enough for correcting for hardware time drift, for instance, but is good enough to correct for one or more hours shift in the timestamps. This verification is performed on all of our datasets before ingesting them into our data management system.

### 4.3. Data Format Variation

One burdensome problem of dealing with data from different types of equipment is handling changing data formats or a variety of possible formatting errors.

The first of such types of problems to be addressed are changes in the data format made by the equipment manufacturer. A considerable amount of format changes from manufacturers are not documented adequately with new versions or software updates. Unfortunately, this type of problem needs to be addressed case-by-case.

One problem that was surprising to us is that some types of failures in the sensors themselves can generate errors in the data format by, for instance, changing the number of data columns in a record. As an example, this could make a record that should have three data columns (e.g., readings on temperature, humidity and solar radiation) actually have extra or missing columns. Similar effects can be caused by connector designed to be generic and support different sensors: a sensor behaving in some unexpected way may cause the data collection node or the logger to perform incorrect conversions or even generate software errors that will affect the data format. In wireless equipment, we have also seen the data format being drastically changed by problems in the transmission of the data. In the presence of radio interference, usually created by the operation of higher powered wireless equipment in proximity of the deployment, the data transmission gets compromised, generating errors in both the values of the readings and the structure of a record.

All these types of errors can cause failures in the ingestion software or, worse, have errors introduced in the data ingestion process without any warnings. Our solution to this was to make ingestion software monitor for format changes and generate informative error messages, allowing identifying problems before ingestion.

### 4.4. Provenance

Given the tailored nature of data pre-processing steps described in this section, it is difficult to keep standardized provenance information and even harder to automate collection of this type of information, as also made evident in [40]. In our current data management solution, simple free text description fields are used to keep track of pre-processing steps and choices. Nonetheless, with a flexible metadata specification tool, such as the one created for our partner project GeoChronos (http://www.geochronos.org/) [56], it would be possible to add specific fields to document evolving aspects of the pre-processing steps.

Although difficult to obtain and maintain, documentation of the pre-processing steps are important to identify not only errors in the pre-processing itself, but also problems with the deployments. For instance, the appearance of too many erroneous records from a wireless data collection node are potential evidence of problems with the sensors, the sensor connections, the node hardware or radio interference sources in the surroundings. The latter problem might even indicate affected readings from other nodes that would otherwise go unnoticed.

## 5. Web-Based Data Analysis

A resourceful and easy to use data management system is the last piece of our solution for large scale *in-situ* monitoring. The pre-processing step presented in the previous section allows the data to be ingested into the system, being stored in an integrated representation. Then users can interact with the system having access to data filtering, aggregation and other more specialized processing operations. Data visualization and retrieval are offered for data at all processing levels after pre-processing, providing a flexible mechanism for analyzing the data within the system or using other tools with the data already narrowed down to the parts of interest. This section discusses these issues, also considering data quality and user interaction aspects.

### 5.1. Uploading Data

The task of data ingestion can be automated for datasets that require pre-processing steps known beforehand. Automated data ingestion methods are particularly useful with deployments that have automated data retrieval, as is the case for data retrieval using a satellite up-link and an the respective Internet service for getting the data. However, new datasets or datasets that needed specialized pre-processing before getting ready to be ingested need a flexible mechanism to map available data to the integrated representation of the data in the system. Properly handling errors and exceptions in the data ingestion processing is necessary from both user experience and data quality perspectives. Automated data ingestion needs timely error generation so the user responsible for the deployment is kept informed and and can take corrective action. Informative descriptions of errors helps the user identify and diagnose the error causes, which is particularly important for manual ingestion of data that was pre-processed in any non-standard way.

Another aspect to be considered is the documentation of the process for every dataset upload. Metadata regarding date and time, data source, user, pre-processing options, among others, help identify sources of error such as faulty time related correction or application of outdated pre-processing methods. Most of these metadata can be collected automatically by the system, which unburdens the user and prevents missing information from less thorough users.

Only with an integrated data representation model it is possible to offer a common user interface, types of filters, aggregation options and any other operation for manipulation of data. Data from different instruments, deployment configurations, retrieval strategies, *etc.*, need to be stored uniformly so all the system’s features are available for all datasets.

### 5.2. Interactive Filters and Operators

**Figure 4.**
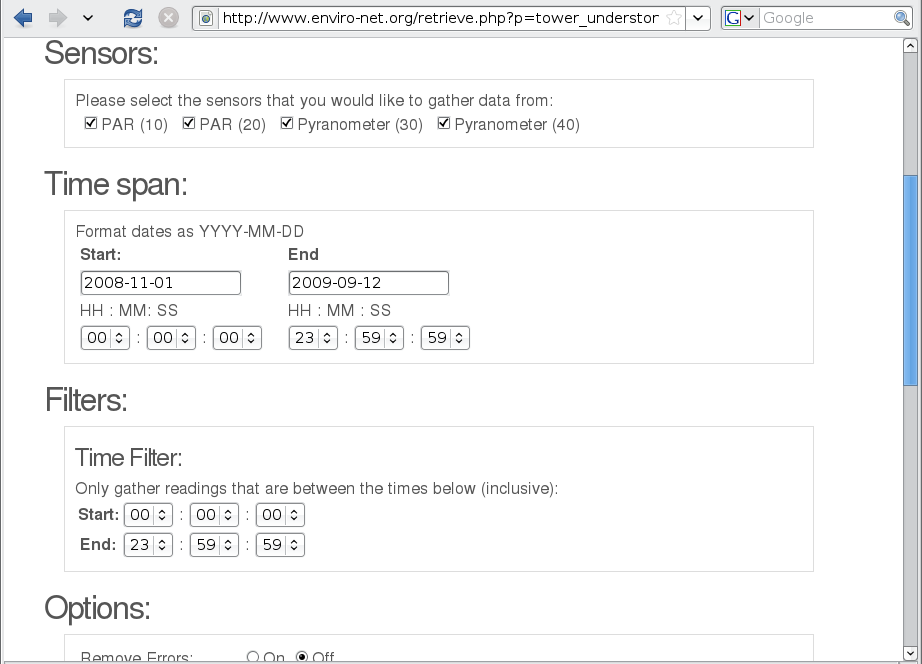
Data retrieval options.

Having the data uniformly stored in a repository, the users can start tailoring datasets to their needs. The most basic functionality to allow this tailoring is being able to apply filters (e.g., only data within a range of values or with low error rates) and aggregation operators (e.g., showing daily or monthly values) to the datasets. Without adequate computational support, many researchers spend days to weeks in this trivial task. Figure 4 shows our interface for a few of these filters to achieve the target data, from the top: which sensors to include, which time span to consider, and which times of the day are of interest. The screen shown is to extract and download a dataset to be used with other tools. Several other options are also available, including filtering out errors, showing raw values (e.g., of voltages, electrical current, or unconverted pulse counts), file and content formats, including data derived from the sensor readings, among others.

Offering quick and easy access to the (corrected and quality checked) sensor readings from a deployment is one of the most essential features of our solution. However, also having data that can be derived from from these readings as easily accessible is what shows the actual potential for data management systems like ours. Our current implementation offers the automatic calculation of vegetation indexes, NDVI and EVI, from solar radiation flux sensors using different methodologies [46–49]. Other products are currently being integrated into Enviro-Net, including LAI, *Vapour Pressure Deficit* (VPD), spatial distribution for *Fraction of Absorbed Photosynthetically Active Radiation* (fAPAR).

### 5.3. Visualization

**Figure 5.**
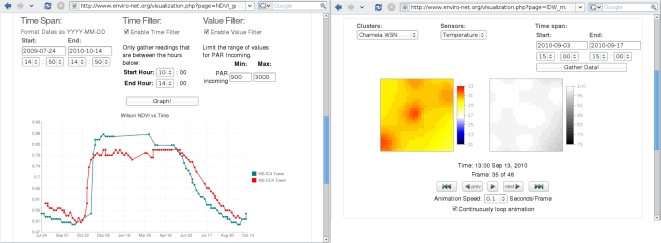
Visualization of derived NDVI (left) and spatial distribution of temperature and its reliability (right).

After tailoring a dataset to specific goals, adequate visualization tools allow easier understanding of events and trends within the monitored areas. The most basic visual tool is graphing the sensor readings of different variables, allowing visual comparison and insight on the measurements in one deployment. However, two features in our web system proved to be invaluable: graphing of datasets that went trough transformations (filtering, aggregation and derived data) and graphing across deployments. These graphing options are depicted on the left side of Figure 5, which shows derived NDVI (using the methodology in [48]) for two different deployments in the Mata Seca State Park, in Brazil, within a specific time span, using only readings close to midday (between 10:00 AM and 2:00 PM local time), filtering out seemingly cloudy days (*i.e.*, including only data records when the measured incoming PAR is more than 900 microeinsteins per second per square meter—*μ*E/m^2^/s), and aggregating the data in daily averages.

The right side of Figure 5 shows another type of visualization strategy based on the spatial distribution of the readings. The graph on the left site uses a color scale to represent variation temperature across an area covered by 12 temperature sensors in the *Chamela Reserve* in Mexico. The graph on the right shows the coverage of the installed sensors (indicating the reliability of the scale), highlighting sensor failures when these occur. Within the specified time span, the system generated a sequence of images which are animated using the controls at the bottom to show the evolution of the temperature and reliability distributions through time. This is a useful tool to observe cyclic (e.g., diurnal or seasonal) changes in the monitored areas.

## 6. Concluding Remarks

This paper presented the Enviro-Net Project, which addresses a variety of issues related to *in-situ* (or ground-based) monitoring of ecosystems, from the deployment of sensors to the delivery of processed data products. A combination of factors make this project unique: (i) acquisition of data at ecosystem level with high spatial and temporal resolutions; (ii) long term, ground-based monitoring; (iii) use of heterogeneous, commercially available, and inexpensive equipment, including wireless sensing technologies; and (iv) integrated data management solution, with a Web-based user interaction with data products.

This scenario, which is increasingly being adopted by other research projects, is described in detail in the paper, discussing lessons learned and pointing out aspects that require attention and could go unnoticed before deployment efforts are well underway. The paper examines not only technical issues of deploying ground-based sensor systems, but also the logistics behind execution and maintenance of deployments, issues related to data retrieval, verification and quality, and publication of data products. The paper discussed evidence that this kind of research was needed, integrating solution to from a number of research efforts and offering a real solution in-situ long term environmental monitoring at high resolution temporal and spatial.

Current efforts include: improving our deployment protocols to deal with arising problems and simplifying the maintenance related tasks; extending our data management system in order to handle larger amounts of data; and adding new data manipulation operations to offer more derived data products. As future work, we intend to focus on data provenance visualization issues, to improve understanding of how data products were generated and allowing automation of reproducibility. Another aspect to be explored in future releases of our data management system is the integration of remote sensing data (from satellite and airborne instruments) into our common interface [57], allowing analysis and comparison of these types of data with ground-based data. We also plan to work on implementing programmatic interfaces to allow software-based access to our data by, for instance, using Open Geospatial Consortium protocols. Lastly, we have plans to include monitoring of equipment life expectancy, particularly of sensors and wireless collector node equipment, in order to create better models for maintenance of long term deployments—by, for instance, increasing the precision of required replacement rates for equipment.

## Figures and Tables

**Table 1.** Dataloggers summary.

**Logger Model**	**Connectivity**	**Storage Memory**	**Power (Battery Type)**	**Est. Longevity^(a)^**
Onset U30	wired data and setup	512 KB	Int. (4.5 or 10 Ah, 4 V) + Solar	solar panel^(b)^
Onset U12	wired data and setup	43,000 samples (64 KB)	Int. (CR-2032 lithium 3 V)	10–12 months
Onset Micro Station	wired data and setup	512 KB	Int. (4 x AA 1.5 V)	10–14 months
Olsonet Collector	wireless data / no setup	256 KB	Int. (2 x AA 1.5 V)	4–5 months
Olsonet Aggregator	wireless data / wired setup	2 GB (remov. SD card)	Ext. (7–12 Ah) + Solar	solar panel^(b)^
Microstrain ENV-Link	wireless data and setup	360,000 samples	Int. (650 mAh) + Ext. (9 Ah)	10–14 months

(a) Estimated longevity with 15 minutes sampling;

(b) Dependent on sun light availability.

**Table 2.** Sensors summary.

**Sensor Model**	**Variable (Unit)**	**Sensor Type**	**Range**	**Accuracy**
Sensirion SHT-75	Temp. (°C)	silicon bandgap	−40.0–123.8 °C	0.3–1.5 °C
	Rel. Hum. (%)	capacitive humidity	0–100% RH	1.8–4.0% RH
Onset S-THB-M00x	Temp. (°C)	silicon bandgap	−40.0–75.0 °C	0.2–0.7 °C
	Rel. Hum. (%)	capacitive humidity	0–100% RH	2.5–4.5% RH
Onset RG3-M	Rainfall (mm/h)	tipping bucket	max 1,270 mm/h	1.00%
Onset S-LIA-M003	PAR (*μ*mol/m2/sec)^(a)^	photons detector	0–2,500 *μ*mol/m2/sec^(c)^	5.0% or 5 *μ*mol/m2/sec
Onset S-LIB-M003	Solar Radiation (W/m2)	silicon photovoltaic detector	0–1,280 W/m2^(d)^	5.0% or 10 W/m2
Apogee SQ-110	PAR (*μ*mol/m2/sec)^(a)^	photons detector	0–2,000 *μ*mol/m2/sec^(c)^	5.00%
Apogee SP-110	Solar Radiation (W/m2)	silicon photovoltaic detector	0–1,100 W/m2^(d)^	5.00%
Decagon ECH2O EC-5	Soil Moisture (VWC^(b)^)	70 MHz capacitance/frequency	0–100 % VWC	1.0–3.0 % VWC

(a) Photosynthetically Active Radiation;

(b) Volumetric Water Content;

(c) For wavelengths between 400 and 700 nm;

(d) For wavelengths between 300 and 1,100 nm.
